# Caffeic Acid Expands Anti-Tumor Effect of Metformin in Human Metastatic Cervical Carcinoma HTB-34 Cells: Implications of AMPK Activation and Impairment of Fatty Acids De Novo Biosynthesis

**DOI:** 10.3390/ijms18020462

**Published:** 2017-02-21

**Authors:** Malgorzata Tyszka-Czochara, Pawel Konieczny, Marcin Majka

**Affiliations:** 1 Department of Radioligands, Faculty of Pharmacy, Jagiellonian University Medical College, Medyczna 9, 30-688 Krakow, Poland; 2 Department of Transplantology, Faculty of Medicine, Jagiellonian University Medical College, Wielicka 258, 30-688 Krakow, Poland; p.konieczny@uj.edu.pl (P.K.); mmajka@cm-uj.krakow.pl (M.M.)

**Keywords:** 5′-adenosine monophosphate-activated protein kinase (AMPK), Metformin, caffeic acid, cervical cancer, metabolic reprogramming

## Abstract

The efficacy of cancer treatments is often limited and associated with substantial toxicity. Appropriate combination of drug targeting specific mechanisms may regulate metabolism of tumor cells to reduce cancer cell growth and to improve survival. Therefore, we investigated the effects of anti-diabetic drug Metformin (Met) and a natural compound caffeic acid (*trans*-3,4-dihydroxycinnamic acid, CA) alone and in combination to treat an aggressive metastatic human cervical HTB-34 (ATCC CRL­1550) cancer cell line. CA at concentration of 100 µM, unlike Met at 10 mM, activated 5'-adenosine monophosphate-activated protein kinase (AMPK). What is more, CA contributed to the fueling of mitochondrial tricarboxylic acids (TCA) cycle with pyruvate by increasing Pyruvate Dehydrogenase Complex (PDH) activity, while Met promoted glucose catabolism to lactate. Met downregulated expression of enzymes of fatty acid de novo synthesis, such as ATP Citrate Lyase (ACLY), Fatty Acid Synthase (FAS), Fatty Acyl-CoA Elongase 6 (ELOVL6), and Stearoyl-CoA Desaturase-1 (SCD1) in cancer cells. In conclusion, CA mediated reprogramming of glucose processing through TCA cycle via oxidative decarboxylation. The increased oxidative stress, as a result of CA treatment, sensitized cancer cells and, acting on cell biosynthesis and bioenergetics, made HTB-34 cells more susceptible to Met and successfully inhibited neoplastic cells. The combination of Metformin and caffeic acid to suppress cervical carcinoma cells by two independent mechanisms may provide a promising approach to cancer treatment.

## 1. Introduction

Despite the recent development of new anti-tumor therapies, cancer still remains one of the leading causes of death in humans. The malignancy of the cervix is the second most common cancer in women world-wide [1]. The efficacy of conventional cancer treatment is often limited and associated with substantial toxicity affecting the whole organism. Moreover, the urgent challenge of therapy is to limit metastasis of cancer cells in the body. This is extremely difficult due to metabolic advantages developed by tumors compared with normal tissues. The intensively proliferating cancer cells reprogram their metabolism to fulfill high metabolic requirements. The critical aspects of metabolic reprogramming in cancer cells substantially contribute to the Warburg effect, involving changes in the glycolytic pathway. Yet, cancer cells were believed not to have functional mitochondria and oxidative phosphorylation (OXPHOS). The earliest observation of Warburg suggested that cancer cells rely solely on glycolysis to provide ATP, even in O_2_ supply [2]. Recent studies present the metabolism of tumor cells as being much more complex, since most cancer cells have metabolically efficient mitochondria to provide intermediates (such as citrate for fatty acid (FA) synthesis) and cofactors (NADH, NADPH) for anabolic pathways [3]. Yet, the inevitable products of mitochondrial oxidative metabolism are Reactive Oxygen Species (ROS), which play important role in the regulation of numerous cellular events, including apoptosis. Consequently, ROS have been taken as a target for dietary antioxidant application in anticancer treatment [4].

It has recently been shown that cancer cells are sensitive to agents that disrupt energy homeostasis, such as metformin (Met), a dimethylbiguanide used for treatment of diabetes mellitus in humans. In fact, numerous epidemiological studies confirmed that metformin may significantly decrease the relative risk of specific cancer incidence and therefore the use of this drug in anticancer therapies has been considered [5] and even tested in ongoing clinical trials [6]. The precise mechanism by which Met regulates metabolic pathways in cell is not certain, but it was well established that Met may interfere with energy metabolism of cancer cells by inhibition of Complex I of the mitochondrial Electron Transport Chain (ETC), reducing oxygen consumption and decreasing ATP synthesis [3]. Considering the latter effect, the beneficial impact of Met on tumor incidence in humans was attributed to the activation of the main energy regulator in cells, 5′-adenosine monophosphate-activated protein kinase (AMPK). In fact, a protective regulatory function of AMPK was reported against various cancers [7]. Nevertheless, the precise role of AMPK in tumor growth and progression is still unclear, also in cervical cancers.

Caffeic acid (*trans*-3,4-dihydroxycinnamic acid, CA) is a potent polyphenolic antioxidant abundant in coffee, red wine, herbs, berries and fruits—such as apricot, apple, and kiwi—as well as in seeds and cereals. CA is a major representative of hydroxycinnamic acids and occurs in food mainly as chlorogenic acid (5-caffeoylquinic acid, an ester of caffeic acid with quinic acid). CA may exert regulatory effects on ROS signaling in cell and its chemopreventive effects in vitro and in vivo have been reported before, with several mechanisms of action proposed [8,9]. However, also a pro-survival effect of CA mediated through a NF-κB signaling pathway was demonstrated in paclitaxel-treated lung cancer cell lines [10]. The exact role of ROS in CA intracellular action remains unclear and probably depends on specific conditions. Recent findings indicate that CA may have anticancer effects via AMPK activation, and such a mechanism was reported in colon cancer cells in vitro [11], but little is known so far about the action of CA in other tumor cells, especially in the context of neoplastic cell metabolism.

The distant metabolic implications of AMPK activation in neoplastic cells bring up not only changes in glucose processing, but also lipid de novo biosynthesis [12]. In eukaryotic cells, tricarboxylic acid (TCA) cycle generates intermediates for biosyntheses, which are crucial for cell growth, such as citrate, a substrate for FA de novo synthesis. The increased FA generation is a commonly observed metabolic alteration during tumorigenesis, providing supply for cell membrane lipids in rapidly proliferating transformed cells [13]. Based on growing understanding of the role of lipids in malignancy, it was suggested that effective antitumor strategy may aim to restrain the subsequent steps involved in formation of long unsaturated FA, molecules that are essential for the function of cancer cell membranes [12]. Although there is a lack of similarity in molecules, both Met and CA were reported to restrain FA synthesis within several cancer cell lines and cause tumor cell proliferation suppression [14,15].

Along with growing evidence targeting metabolic disturbance of cancer cells, the new strategies to improve the efficiency of anticancer therapies recognize the energetic and biosynthetic aspects of tumor cells metabolism. Therefore, the objective of the novel approach is to develop molecularly targeted anticancer strategies combining specific activities of anticancer compounds in order to improve the outcome of the treatment [3,16,17]. Recent findings suggest that combined therapy may be efficient to increase tumor regression, to prolong the remission of cancer, and to prevent metastasis. In particular, the correct combination of natural compounds having chemopreventive properties and various cytotoxic drugs regulating specific target proteins or whole metabolic pathways appears very attractive [18]. Indeed, a wide variety of natural compounds offer the opportunity to address distinct regulation of metabolism in different types of cancer [19].

In the present paper, we aimed to determine whether Met, CA, and combined treatment of Met and CA may effectively decrease human cancer cell viability in vitro. To achieve the objectives, HTB-34 human cervical cancer cells with aggressive metastatic phenotype were employed. We tested if the combination of Met and CA may enhance cell death in human cervical HTB-34 cancer cell line compared with compounds acting alone. We explored if the possible mechanism of treatment involves the activation of apoptosis and/or necrosis. Since the effect of both compounds on AMPK in HTB-34 human cervical cancer cells has not been studied before, we tested how treatment of HTB-34 cells with CA, Met, or CA/Met implicates the action of AMPK by measuring the phosphorylation of this enzyme and its protein ACC-1 (involved in FA synthesis) downstream. In the context of targeting metabolic alterations of cancer cells, it was interesting to explore how Met and CA interfere with metabolism of tumor cells, especially with glucose processing and TCA cycle supply via oxidative decarboxylation of pyruvate and also by glutamine funneling to the cycle. As a TCA cycle product, citrate serves as a precursor for lipid formation. We focused on the effect of Met and CA on the regulatory proteins of FA de novo biosynthesis. Since the reprogramming of glucose and lipid metabolism in cancer cells is associated with oxidative stress in mitochondria, we assessed if enhanced ROS production may be involved in cell death caused by Met and CA action.

## 2. Results

### 2.1. Co-Treatment of HTB-34 Cells with Met and CA Potentates Cell Death via Apoptosis, Inhibits Proliferation of Cells and Induces Changes in Cell Cycle

**Figure 1:**
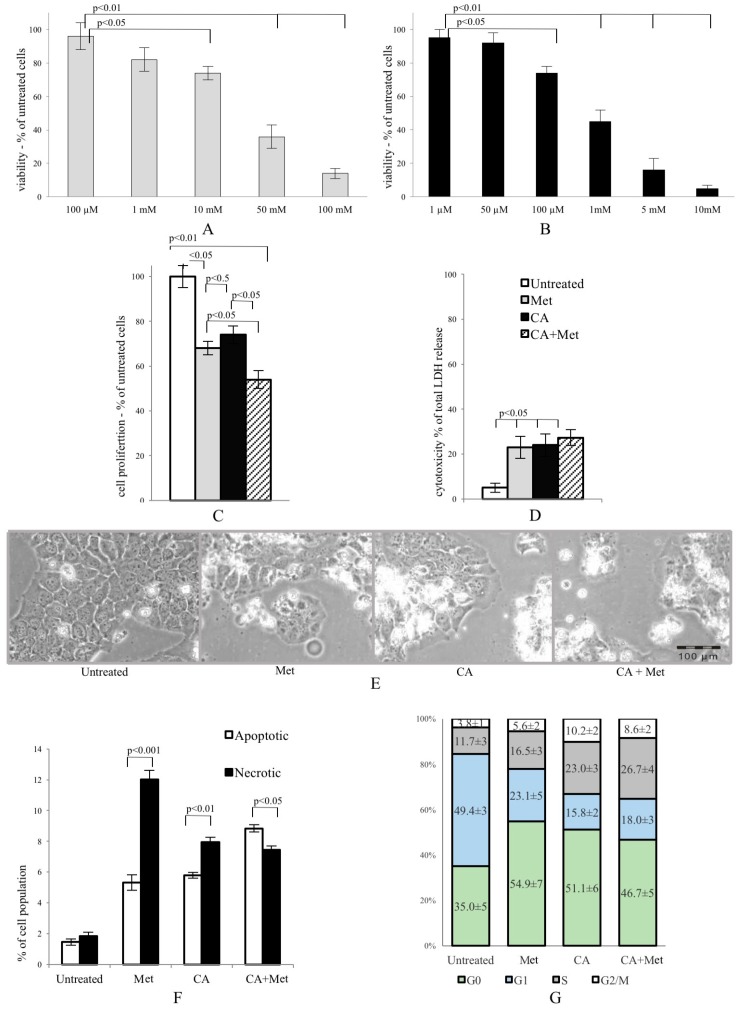
Metformin (Met) and caffeic acid (CA) exert an anti-proliferative effect on HTB-34 human cervical cancer cells. Sensitivity of HTB-34 to Met ((**A**) 100 μM to 100 mM) and CA ((**B**) 1 μM to 10 mM) after 24 h treatment as measured with MTT assay. Effect of Met and CA treatment on (**C**) cell proliferation and (**D**) LDH release; (**E**) Cell culture morphology under phase contrast light microscope after CA (100 µM), Met (10 mM), and Met/CA treatment. The detrimental effect caused by Met and CA alone is mainly due to necrosis, while combination of Met and CA significantly increase apoptosis in cancer cells (**F**) followed by a shift towards S and G2/M phases of cell cycle in population of treated cells (**G**). Experiments were repeated three times with similar results.

Met and CA inhibited proliferation of HTB-34 cells, as shown in Figure 1A,B, respectively. We found that 100 µM treatment of CA for 24 h inhibited proliferation of cells compared with untreated control (*p* < 0.05 vs. control). Similarly, the presence of 10 mM Met in culture medium also significantly deceased viability of cancer cells (*p* < 0.05 vs. control) and enhanced cytotoxicity (*p* < 0.05 vs. control) (Figure 1D). The exposition of HTB-34 cells to CA in the presence of Met caused greater reduction of cell proliferation compared to CA alone (*p* < 0.05) and Met alone (*p* < 0.05, Figure 1C) and aggravated cell morphology (Figure 1E), observed under phase contrast microscope. Met/CA co-treatment significantly induced apoptosis compared to necrosis (*p* < 0.05) (Figure 1F). These changes were followed by a shift in cell population towards S and G2/M phases of cell cycle after CA and Met/CA treatment (Figure 1G phase S: *p* < 0.01 for CA vs. untreated control, *p* < 0.05 for Met/CA vs. untreated control; phase G2/M: *p* < 0.01 for CA vs. untreated control, *p* < 0.05 for Met/CA vs. untreated control).

### 2.2. CA Activates AMPK, Changes the Activity and Expression of Enzymes Involved in Glucose Catabolism, Inhibits Glucose Uptake and Lactate Formation in HTB-34 Cells

**Figure 2:**
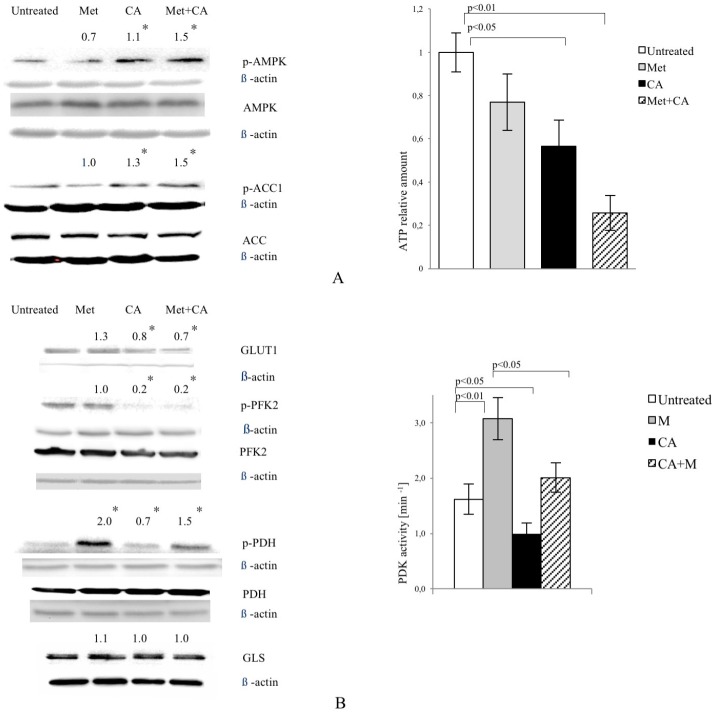

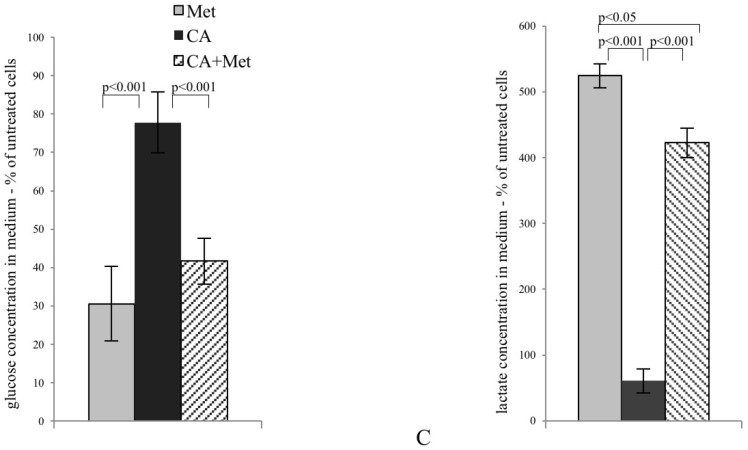
CA activates AMPK in HTB-34 cells along with increasing pyruvate decarboxylation via PDH complex and decreasing glucose consumption and lactate production. Immunoblot analysis (the details described in Materials and Methods) reveals enhanced phosphorylation of AMPK on T^172^ residue by CA alone and Met/CA co-treatment along with activation of AMPK downstream ACC-1 and decrease of ATP content (**A**); Met and CA have slight, opposite effect on glucose uptake via GLUT-1. CA and Met/CA cause loss of PFK-2 activity. CA increases PDH-E1α phosphorylation on S^293^ and inhibits PDH kinase (PDK) activity facilitating pyruvate flux via PDH complex. Note the opposing effect of Met on PDH phosphorylation (caused by PDK activation) compared with CA and recovery of metformin-inhibited PDH complex by co-treatment with CA. CA, Met, and Met/CA treatment has no effect on Glutaminase (GLS) expression (**B**); For Western blot analyses β-actin was used as the protein loading control, band intensities were quantified by densitometry analysis and expressed relative to the control (* *p* < 0.05 vs. untreated control). CA decreases glucose consumption and lactate release into culture medium (**C**) after 24 h of incubation and attenuates the effect of Met. CA was used at 100 µM and Met at 10 mM for 24 h. Experiments were repeated three times with similar results.

As shown in Figure 2A, CA activated AMPK in HTB-34 cells, while Met failed to phosphorylate the enzyme. CA also phosphorylated Acetyl-CoA carboxylase 1 (ACC1) at S^79,80^. The similar effect was measured in cells exposed to CA/Met. ATP content was decreased in cells exposed to CA and Met/CA. CA downregulated glucose transporter GLUT1 expression alone and as co-treatment with Met (Figure 2B). CA and CA/Met treatment decreased Phosphofructokinase 2 (PFK2) activity by its dephosphorylation on S^466^ residue. To examine the effect of Met and CA on the process of oxidative decarboxylation, the phosphorylation (deactivation) of Pyruvate Dehydrogenase Complex (PDH) at S^293^ by the action of Pyruvate Dehydrogenase Kinase (PDK) was assessed. The activation of PDH caused by CA was followed by significant decrease in PDK activity (*p* < 0.05 vs. untreated control). Met inhibited PDH activity and caused significant rise in PDK activity (*p* < 0.01 vs. untreated control). CA, when co-treated with Met, antagonized its effect on PDH phosphorylation and PDK activity. Any changes in expression of glutaminase (GLS) were not observed (Figure 2B).

The exposition of HTB-34 cells to CA significantly inhibited glucose consumption (Figure 2C, *p* < 0.001 vs. Met, *p* < 0.001 vs. CA/Met) and substantially decreased the lactate level in medium when compared to the effect of Met (Figure 2C, *p* < 0.001 vs. Met, *p* < 0.001 vs. CA/Met). The co-treatment of HTB-34 cells with Met and CA significantly limited lactate excretion compared with Met-treated cells (*p* < 0.05 vs. Met).

### 2.3. CA Augments Mitochondrial Oxidative Stress

**Figure 3:**
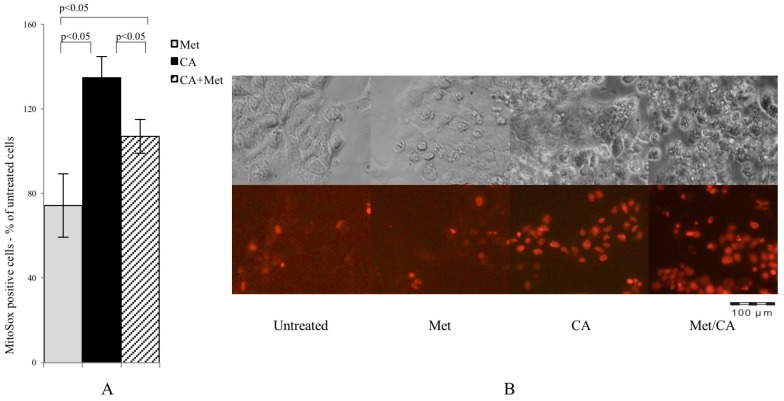
CA and Met/CA causes significantly enhanced generation of mitochondrial ROS measured with MitoSox Red probe by flow cytometry (**A**) and visualized by fluorescence microscopy after incubation of HTB-34 cells with Met at 10 mM and CA at 100 µM for 24 h; (**B**) note the induction of mitochondrial oxidative stress after incubation of cells with CA and Met/CA (red fluorescence indicate mitochondrial superoxide). Experiments were repeated three times with similar results.

In present experiments mitochondrial superoxide formation was measured by MitoSox staining followed by cytometry analysis. As shown in Figure 3A, CA was a most potent ROS inducer and the effect was preserved after co-treatment with Met, while Met itself caused no effect (*p* < 0.05 for CA vs. Met, *p* < 0.05 for CA vs. CA/Met), as also shown on microphotographs (Figure 3B).

### 2.4. Met Treatment Attenuates Fatty Acids (FA) De Novo Synthesis by Decreasing of Expression of Regulatory Enzymes

**Figure 4:**
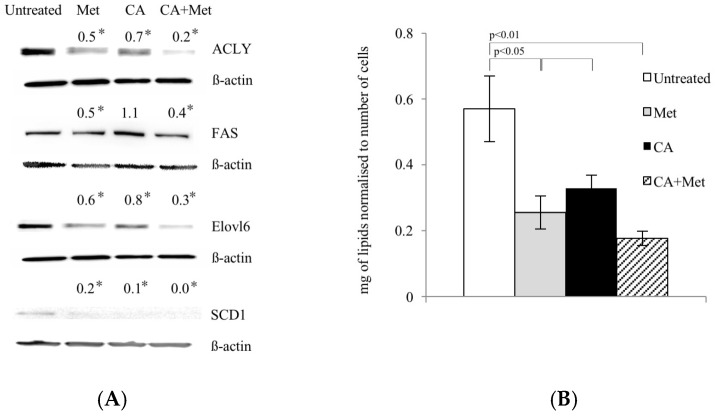
Met decreases expression of lipogenic genes (**A**) and unsaturated FA content (**B**) in human cervical carcinoma HTB-34 cells. Total cell lysates were subjected to SDS-PAGE followed by Western blot analysis and chemiluminescent detection (band intensities were quantified by densitometry analysis; the details described in Material and Methods, * *p* < 0.05 vs. untreated control). Note that co-treatment of cells with Met and CA for 24 h restrains the expression of ACLY, FASN, ELOVL6, and SCD1 (**A**) and significantly decreases unsaturated FA content in cancer cells, as measured by spectrophotometric assay. Data shown here are from a representative experiment repeated three times with similar results and presented as mean values ± SD (**B**).

During energy depletion, AMPK inhibits FA synthesis not only by direct phosphorylation and inactivation of ACC1, but also by exerting the effect on regulatory enzymes of the pathway at the level of gene expression [11,12]. Therefore, we investigated whether Met and CA may decrease FA de novo synthesis by downregulation of enzymes: ATP Citrate Lyase (ACLY), Fatty Acid Synthase (FAS), Stearoyl-CoA Desaturase-1 (SCD1), and Fatty Acyl-CoA Elongase 6 (ELOVL6). Met caused a decrease in protein amounts of all enzymes, as shown in Figure 4A. The quantitation of total unsaturated lipids in HTB-34 cells exposed to compounds revealed the same pattern, since Met alone and as co-treatment with CA significantly decreased the amount of FA (*p* < 0.05 for Met vs. untreated control, *p* < 0.01 for Met/CA vs. untreated control) (Figure 4B).

## 3. Discussion

Since metabolic reprogramming of tumors may cause a selective advantage for survival of transformed cells, the recognition of the role of master regulatory enzymes in the metabolic network is of particular interest. It has recently been suggested that the regulation of AMPK by biguanides and by naturally occurring compounds may provide potential therapeutic benefits [5]. Extending these findings, we aimed to assess if the intervention in lipid biosynthesis and the regulation of metabolic pathways within neoplastic cells via AMPK and TCA cycle supply may be a potent strategy for improving cancer treatment efficacy [20]. Despite extensive ongoing research, the function of key metabolic regulators in cancer has only been partially understood. To analyze the action of Met and CA in cervical tumor, human HTB-34 cancer cells were used. Recently both compounds, CA and Met, were independently reported to activate AMPK and to exert anti-cancer effect via AMPK-dependent mechanisms in other cancer cell lines in vitro [14,15].

In most cancer cells, increased glucose uptake and enhanced glycolysis to lactate provides energy and contributes to the rapid growth and cellular division typical for cancer cells. In the present study, CA alone and applied with Met reduces the expression of tumor cell glucose transporter, GLUT1. Cancer cells very effectively supply glucose even at its low concentration in bloodstream, as Km value of GLUT1 transporter is about 1 mM. Natarelli et al. reported that CA has no effect on GLUT1 expression and membrane localization in normal endothelial cells, which might suggest selective effect of CA in neoplastic cells, but more extensive study is necessary [21]. CA, also unlike Met, reduces the activity of PFK2, an enzyme regulating the rate of glycolysis. The decrease of PFK2 activity, together with GLUT1 downregulation caused by CA, probably slows down the rate of glycolysis in HTB-34 cells. The effect is preserved after Met/CA treatment, revealing opposite metabolic action of both tested compounds, CA and Met. The aggravation of glucose catabolism is reflected in the reduced amount of lactate produced by cells due to the exposure to CA. The boosted lactate production was reported as primary metabolic change caused by Met in tumor cells [15], which is also confirmed by our observations. On the other hand, Met exerted antitumorigenic effect in cervical carcinoma HeLa and SiHa cells by decreasing the expression of a limiting glycolytic enzyme Pyruvate kinase muscle isozyme M2 (PKM2) involved in mTOR-dependent pathway regulating epithelial-mesenchymal transition (EMT), as reported by Cheng et al. [22].

Along with vigorously promoted transformation of pyruvate to lactate in cancer cells, TCA cycle metabolites are strongly decreased [23]. In our study, CA—unlike Met—substantially contributes to fueling the TCA cycle with pyruvate. The enhanced intermediate entry to TCA cycle occurs due to increased PDH activity. Mitochondrial PDH complex catalyzes the irreversible oxidative decarboxylation of pyruvate to acetylCo-A. PDH is a key regulatory point of metabolic pathways, which are determinant for the overall glucose and lipid disposal within the cell. The activity state of PDH is precisely regulated by the covalent modification via specific kinase (PDK), which in turn was reported as a subject to anticancer intervention [20,24]. In present experiments, CA inhibits PDK activity in HTB-34 cells and the effect is opposite to that of Met. What is more, when cells are exposed concomitantly to both compounds, CA attenuates the action of Met by stimulating oxidative decarboxylation via PDH/PDK. Previously, the phenethyl ester of caffeic acid (CAPE) was reported to modulate PDK1 at the level of its gene expression in a context of induction of hypoxia via hypoxia-inducible factor 1α (HIF1α), which might have influenced cancer cell adaptation to metabolic demands [25].

The facilitated glutamine catabolism is an additional important source for energy production in tumor cells, besides glucose. While carbon flux via oxidative decarboxylation from pyruvate is limited, glutamine provides carbons to feed the TCA cycle, and GLS serves as a key regulator of this substrate entry to TCA [26]. Such metabolic adaptation of neoplastic cells may be problematic, because even when glucose supply is decreased, glutamine may rebalance the metabolism and provide a carbon flux for biosynthesis. Here, we did not observe any changes in GLS expression due to CA or/and Met treatment. The TCA cycle has not been additionally supplied with metabolic intermediates via GLS. Our data are consistent with findings of Janzer et al. [23] that glutamine uptake is not changed by biguanides action in cancer cells.

**Figure 5:**
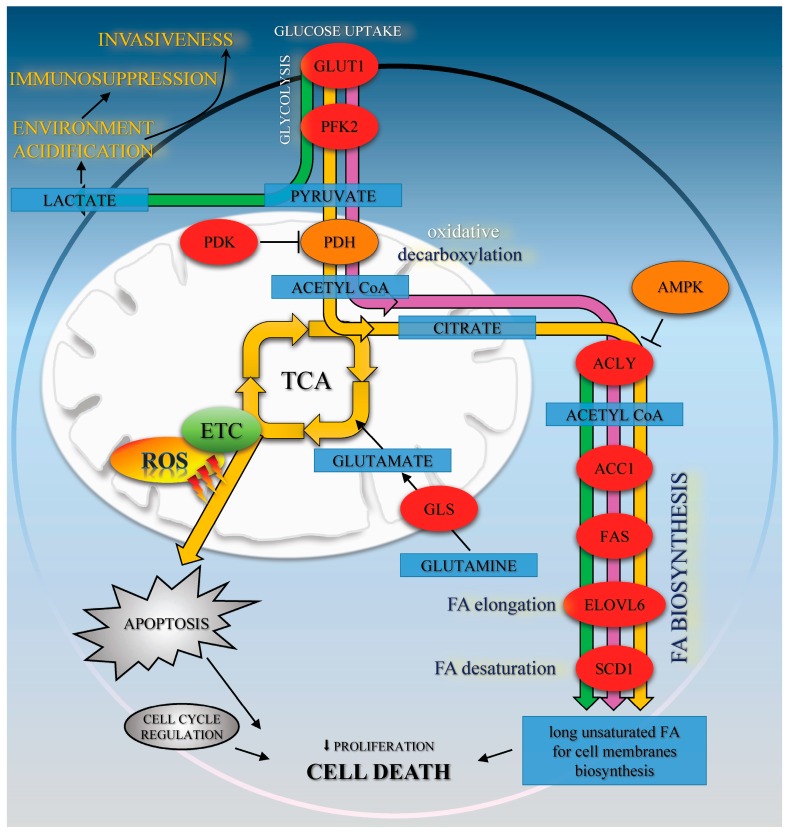
The effects of Met (green path), CA (yellow path), and Met/CA co-treatment (purple path) on oxidative metabolism and fatty acids biosynthesis in HTB-34 cells. Met promotes glycolysis to lactate and regulates FA de novo synthesis. CA activates AMPK, enhances oxidative metabolism of glucose via PDH and ROS formation in mitochondria and apoptosis. Met/CA activates AMPK, inhibits FA biosynthesis in the short-term via ACC1 and in the long-term by downregulation of ACLY, FAS, ELOVL6, and SCD1 enzymes expression.

The enhanced FA synthesis provides lipids for membrane biogenesis and promotes the growth and survival of neoplastic cell, playing a key role in the metabolic phenotype of tumor cells [13]. Our data show that CA co-treatment with Met activates AMPK and suppresses its downstream enzyme, ACC1, involved in lipid biosynthesis. Therefore, it was of interest to find out if tested compounds may interfere with lipid content within the cells. We measured that Met and CA decrease the total amount of unsaturated FA in cancer cells. To explore the mechanism of that observation, we focused on the expression of subsequent regulatory enzymes involved in FA synthesis (ACLY, FAS), elongation (ELOVL6) and desaturation of FA (SCD1). In tumors, the overexpression of those regulatory enzymes was linked to the fact that neoplastic cells tend to be invasive and metastatic [12]. In our experiments, the greatest downregulation of ACLY, FAS, ELOVL6, and SCD1 proteins expression was measured after treatment of cells with Met and Met/CA suggesting that major effect on FA synthesis impairment is exerted by Met. Indeed, Met was reported before to alleviate intracellular FA synthesis [11] and that metabolic alteration was attributed to inhibition of mitochondrial respiration caused by the drug [10]. It may also be observed that the impaired FA biosynthesis in HTB-34 cells is a consequence of restrained funneling of the TCA cycle via PDH complex and subsequent reduced formation of substrate for FA de novo synthesis caused by Met (Figure 5).

The mechanism of suppression of tumor cells growth due to impaired lipogenesis, involving activation of AMPK and blocking of its downstream protein ACC1, was reported before and such activity of Met was studied in several cancer cell lines [7,27]. Griss et al. showed that Met reduced production of metabolic intermediates of TCA cycle and suppressed FA de novo synthesis, which resulted in decreased proliferation of non-small cell lung cancer H1299 cells [28]. Chiang et al. reported that augmentation of AMPK cascade and subsequent inhibition of FAS expression caused by exposition of colon cancer CRC cells to CA derivatives inhibited the growth of cells [14]. The results presented by Hwang et al. indicated that Chlorogenic Acid derivatives isolated from *Salicornia herbacea* may exert cytotoxic effect on and decrease proliferation of human Hep G2 cells, including AMPK activation and resulting impaired expression of SREBP-1c and FAS proteins [29]. A similar mechanism was reported in Hep G2 cells for other polyphenolic compound, Epigallocatechin Gallate (EGCG) [30]. Taking into consideration the anti-proliferative activity of CA and Met involving impaired lipid metabolism, we can speculate that the alleviation of FA biosynthesis due to Met action and the restoration of PDH caused by CA may result in the impairment of proliferation and survival of cervical carcinoma HTB-34 cells. What is more, the regulation of metabolic events combined with modulation of the cell cycle by Met was suggested to suppress the growth of several neoplastic cell lines [28]. Cai et al. reported that Met at a concentration of 10 mM inhibited proliferation and induced G0/G1 phase arrest in human esophageal squamous cell carcinomas [31]. Also, our study revealed that exposition of HTB-34 cells to Met increased the population of cells in G0/G1 phase. CA at a concentration of 80 µM was recently reported by Murad et al. to decrease cell viability by stimulating apoptosis in human colon adenocarcinoma HT-29 cell culture and by promoting specific changes in cell cycle regulation [8]. Kuo et al. showed that cell proliferation of TW2.6 human oral squamous carcinoma cells was suppressed by caffeic acid phenethyl ester through a decrease in G1 phase cell population with concomitant increase in G2/M phase and enhanced apoptosis, which was consistent with our findings considering cells exposed to CA and CA/Met. Kuo et al. attributed the effect to the attenuation of G2/M checkpoint, essential for DNA damage repair [32]. It may be presumed that enhanced ROS, produced within cells due to exposition to CA, may promote DNA damage instead of restoration and cumulating effect of aberrations may lead to cell death. ROS are vigorously produced by mitochondrial ETC as a consequence of oxidative metabolism and active TCA cycle. It was well established that in cancer cells the excessive amounts of ROS may accumulate and trigger death program (Figure 5). Recently, it was found that AMPK activation may be linked to ROS production [33,34]. It was also suggested that anticancer activity of CA is due to its ability to regulate ROS-induced cell death, but the mechanism is complex and depend on cellular context [7,8]. However, our data show significant connection of pyruvate funneling into TCA cycle and enhanced ROS formation after treatment of HTB-34 cells with CA. Further experiments would help to elucidate the involvement of ROS into mechanism of CA action in HTB-34 cells.

In our study, concerted action of both compounds, CA and Met, exerts an anti-proliferative effect on cervical carcinoma HTB-34 cells, followed by the decrease in the number of cells. Co-treatment of cells with Met and CA results in apoptosis as a supreme way of cell death and the effect is greater than that of CA and Met applied alone. At the same time, the inhibition of cell growth caused by Met is mainly due to necrosis. On the basis of the results obtained, one cannot unambiguously elucidate why apoptosis increases in HTB-34 cells exposed to Met/CA. However, our data suggest that the employment of both compounds to suppress cervical carcinoma cells may provide a promising approach for cancer treatment. On the other hand, the main problem with natural dietary products is that effective, usually micromolar, concentrations of bioactive compounds are rather transient in humans. Plasma concentration of CA has been reported rarely to exceed 1 µM after the consumption of 10–100 mg of a single compound in humans [35], while Met occurs at 10 mM in human plasma and that level may be sustained much longer [9,15,36]. Therefore, in our study we focused on cervical cancer as an experimental model, since the location of tumor within the body might provide the opportunity to use the bioactive compound as a topical formulation, bypassing the problem of low CA bioavailability in bloodstream. The studies on cellular uptake of CA by cancer cells report that phenolic acids are absorbed and metabolized by cancer cells [11]. Accumulating evidence addresses the application of AMPK activators, such as Met and CA, in medical treatment of several metabolic disorders including cancer [8]. Moreover, the emerging data highlight mitochondrial metabolism as essential for tumorigenesis. Consequently, the combination of two or more compounds, regarding different mechanisms of action on metabolic pathways and low toxicity in humans, is of interest.

In our experiments, we aimed to study the influence of compounds on metabolic reprogramming of late-stage invasive cervical carcinoma cells. Therefore, the aggressive metastatic HTB-34 line with epithelial morphology has been selected due to advanced malignant transformation of the cells. HTB-34 cells were obtained from secondary cervical tumor and they had undergone epithelial to mesenchymal transition (EMT) and subsequent mesenchymal to epithelial transition (MET). The data obtained from only one particular cancer cell line must be interpreted with caution, since the regulation of biochemical pathways in various cervical carcinoma cell lines may differ remarkably depending on stage of tumor and epithelial/mesenchymal characteristics.

We conclude that, in cervical carcinoma HTB-34 cells, CA modestly activates AMPK, while Met exerts its effect on FA biosynthesis by downregulation of several enzymes involved in the de novo synthesis of long unsaturated FA, such as ACLY, FAS, ELOVL6, and SCD1. The alleviation of FA synthesis caused by Met due to a reduction in citrate for fueling of FA biosynthesis probably plays a significant role in the restraining of the proliferation rate and promoting cell death in cervical cancer cells exposed to Met. CA mediates the metabolic reprogramming of HTB-34 cell by enhancing of glucose processing via oxidative decarboxylation. The increased oxidative stress, as a result of CA treatment, sensitizes cancer cells and make HTB-34 cells more susceptible to Met. Our data suggest that CA and Met induce cell death in human cervical metastatic cells by two independent mechanisms, CA by increasing ROS production and Met by suppressing of substrate formation for FA biosynthesis. The treatment of neoplastic cells with both compounds provides a promising approach to evoke metastatic cervical HTB-34 cells death.

## 4. Materials and Methods

### 4.1. Cell Culture and Incubation Conditions

Human cell line HTB-34 (ATCC designation MS751) was derived from the American Type Cell Culture collection. Cells were maintained as a monolayer culture in EMEM-Eagle’s Minimum Essential Medium (Lonza, Walkersville, MD, USA), supplemented with 10% *v*/*v* FBS (Eurex Sp z o.o., Gdansk, Poland) and 50 µg/mL of gentamicin, at 37 °C in a humidified atmosphere of 5% CO_2_ and passaged twice a week. For experiments, HTB-34 cells at a density of 1.5 × 10^5^ cells/mL were placed in cell culture plates (Sarstedt, Numbrecht, Germany) and incubated to reach adequate confluency. Then cells were washed with PBS solution (Lonza) and kept for 24 h in EMEM-containing antibiotic and 0.5% *v*/*v* of bovine serum albumin (BSA, Sigma-Aldrich, Seelze, Germany). This step was taken in attempt to silence cells, since the amount of insulin in serum-containing medium may be higher compared with cells in vivo. Then the medium was replaced with a new EMEM/0.5% BSA with adequate volumes of stock solution of CA (100 µM/L, Sigma-Aldrich) or Met (10 mM, Sigma-Aldrich) or CA (100 µM) and Met (10 mM) and exposition of cells to appropriate compound was continued for 24 h. The 1% (*v*/*v*) of CA solvent, dimethyl sulfoxide (DMSO) was added to an untreated control and to Met containing wells. After incubation, cells and media were collected.

The number of cells was assessed by automatic cell counter Countess (Gibco Laboratories, Gaithersburg, MD, USA). The morphology of cell culture was investigated by an inverted light microscope (Olympus IX-70 microscope with fluorescence, Olympus, Hamburg, Germany). Representative images were obtained at 20× magnification.

### 4.2. Anti-Proliferative Assay

MTT, 3-[4,5-dimethylthiazol-2yl]-2,5-diphenyl tetrazolium bromide was purchased from Sigma-Aldrich, Seelze, Germany. Briefly, 200 μL of a suspension of exponentially dividing cells was placed in each well of a 96-well microtiter plate (Sarstedt, Numbrecht, Germany) in medium. Untreated cells were cultured in medium and solvent (positive control, 100% of growth). The test was conducted as described previously [37]. The absorbance was measured at 570 nm (the reference wavelength was 630 nm) using a microplate reader Infinite M200 Pro (Tecan, Salzburg, Austria).

### 4.3. Cytotoxicity Assay

The cytotoxicity was measured as Lactate Dehydrogenase (LDH) leakage from cells exposed to compounds and determined by using commercially available BIOLABO SAS–L.D.H. (LDH-P) SFBC Modified Method kit according to manufacture protocol (Biolabo S.A., Maizy, France). For LDH test, cells were seeded into a 96-well plate and exposed to drugs as described in 4.1. Afterwards, 24 h of incubation media were collected and cells were lysed (Triton-X, 1% *v*/*v*, Sigma-Aldrich) to release the intracellular LDH. Then NADH concentration generated in the reaction of the reduction of pyruvate was measured at 340 nm using microplate reader (Tecan). For each sample the result was given as the percentage of LDH in the medium versus total LDH activity in the cells.

### 4.4. Cell Apoptosis and Necrosis Quantitation

After incubation cells were collected and analyzed on FACSCanto10C flow cytometer, using BD System Software (BD FACSDIVA V8.0.1, BD Biosciences Immunocytometry Systems, San Jose, CA, USA). Fluorescent dyes Annexin-V (excitation/emission 490/515 nm) and Ethidium homodimer (EthD-III, excitation/emission 528/617 nm) were used (Biotium, Fremont, CA, USA). The cells were gated according to forward (FSC), side scatter (SSC), and appropriate fluorescence parameters. The living cells were defined as negative for Anexin-V and EthD-III, the apoptotic cells consisted of Anexin-V positive/EthD-III negative cells (early apoptosis) and Anexin-V/EthD-III positive cells (late apoptosis); the necrotic cells were Anexin-V negative and EthD-III. The results were given as the percentage of apoptotic or necrotic cells of the total counted cells (Figure S1).

### 4.5. Cell Cycle Analysis

For detection of cell cycle distribution upon different treatments, cells were trypsinized, washed with PBS, and centrifuged for 5 min at 250× *g*. BD IntraSure kit (BD Biosciences) was used for cell fixation and permeabilization, according to manufacturer’s protocol and followed by 15 min of dark incubation in room temperature of cells with Ki-67 antibody (Thermo Fisher Scientific Inc., Waltham, MA, USA). Thereafter, cells were stained with DAPI fluorescent dye (excitation maximum at 360 nm and fluorescence emission maximum at 460 nm, Thermo Fisher Scientific Inc.) and processed for FACS-based cell cycle analysis (Figure S2).

### 4.6. Immunoblot Analysis

Cells for immunoblot analysis were homogenized in M-PER buffer (Thermo Fisher Scientific Inc.) with a protease inhibitor cocktail (Merck, Darmstadt, Germany). The protein samples were resolved via standard SDS-PAGE and then transferred to PVDF membranes. The membranes were blocked with buffer contained 1% BSA in TBST (20 mM of Tris-hydrochloride, pH 7.5, 150 mM NaCl, 0.05% Tween 20; BioRad, Laboratories, Hercules CA, USA) as described previously [38]. Immunoblottings were performed using primary antibodies: anti-AMPK, (Cell signaling, Danvers, MA, USA), anti-p-AMPK (Cell signaling), anti-p-PDH (Abcam, Cambridge, MA, USA), anti-PDH (Cell signaling) anti-GLUT1 (Santa Cruz Biotech., Santa Cruz, CA, USA), anti-PFK2 (Santa Cruz Biotech), anti-p-ACC1 (Cell signaling), anti-ACC1 (Cell signaling), anti-ACLY, anti-FAS (Santa Cruz Biotech), anti-ELOVL6 (Santa Cruz Biotech), and anti-SCD1 (Santa Cruz Biotech), followed by HRP-linked secondary antibody (Santa Cruz Biotech). β-Actin (1:1000, Cell signaling) was the loading control. The specific immunoreactivity was demonstrated by enhanced chemiluminescence (the immunoblots were developed using the Super Signal West Pico Chemiluminescent Substrate Kit, Pierce Chemical, Rockford, IL, USA) using Gel Logic Imaging System 1500 (Kodak; Molecular imaging System Corestea Health Inc., Rochester, NY, USA). Total protein was measured by the Bradford method, as described elsewhere, using BSA as a standard. Each experiment was repeated three times.

### 4.7. Assay of Pyruvate Dehydrogenase Kinase (PDK) Activity

For the Preparation of mitochondria, liver mitochondria were isolated from cells exposed to compounds and prepared as described in [39]. Protein amount was assayed by Bradford method. For the assessment of PDK, mitochondria were incubated for 25 min in KCl medium (120 mM KCl, 20 mM Tris-hydrochloride, pH 7.4, 5 mM K2HPO4, 2 mM EGTA) containing 10 µM of uncoupler carbonyl cyanide-chlorophenylhydrazone to convert inactive PDH complex into active.

For the enzyme assay, PDK activity in mitochondrial extracts was measured according to Wu et al. [39]. For the assay, mitochondrial pellets were disrupted in extraction buffer (50 mM K_2_HPO_4_, 10 mM EGTA, 2 mM DTT, 1% BSA, 0.5% Triton-X100). PDK activity was expressed as the rate of ATP-dependent inactivation of the PDH complex and calculated as the apparent first-order rate constant.

### 4.8. Glucose Consumption and Lactate Production Analysis

HTB-34 cells were seeded into multi-well plates and exposed to compounds for 24 h, as described above, and then culture media were collected. Glucose concentration in media was determined using a colorimetric test (Sigma-Aldrich) and lactate release from cells was determined in media using commercially available kit (Sigma-Aldrich). Both assays were performed according to the manufacturers protocols using Universal Microplate Reader ELX800NB (Bio-Tek Instruments INC, Vinooski, VT, USA) and for both tests, the absorbance was measured at 540 nm.

### 4.9. Measurement of Mitochondrial ROS Level

Mitochondrial ROS were measured using MitoSox Red reagent (Invitrogen, Carlsbad, CA, USA) according to manufacturer protocol using FACSCanto10C flow cytometer (BD Biosciences). MitoSox superoxide indicator has excitation/emission maxima of 510 and 580 nm, respectively. The fluorescence was also observed and recorded by fluorescence microscopy.

### 4.10. Quantitation of Unsaturated Fatty Acids

The extraction of total lipids from cells exposed to compounds was performed using commercially available Lipid Extraction Kit (Cell Biolabs Inc., San Diego, CA, USA) according to the manufacturer protocol.

The content of unsaturated fatty acids in cells was performed using commercially available kit (Cell Biolabs Inc.) according to the manufacturer protocol.

The absorbance was measured at 550 nm using Bio-Tek microplate reader.

### 4.11. Statistical Analysis

The experimental data were shown as mean ± SD. Analysis was performed using one-way ANOVA followed by a Duncan post-hoc test. *p*-values < 0.05 were considered statistically significant. Calculations were carried out using the commercially available package, Statistica PL v.10 (StatSoft, Tulusa, OK, USA).

## 5. Conclusions

In conclusion CA modestly activates AMPK, while Met exerts its effect on FA biosynthesis by downregulation of several enzymes involved in the de novo synthesis of long unsaturated FA, such as ACLY, FAS, ELOVL6, and SCD1. CA and Met induce cell death in human cervical metastatic cells by two independent mechanisms, CA by increasing ROS production and Met by suppressing of substrate formation for FA biosynthesis. The treatment of neoplastic cells with both compounds provides a promising approach to evoke metastatic cervical HTB-34 cells death.

## Supplementary Materials

Supplementary materials can be found at www.mdpi.com/1422-0067/18/2/462/s1.

## Abbreviations

CA	Caffeic Acid
Met	Metfomin
FA	Fatty Acids
ACC1	Acetyl-CoA Carboxylase 1
ACL	ATP Citrate Lyase
FAS	Fatty Acid Synthase
SCD1	Stearoyl-CoA Desaturase-1
ELOVL6	Fatty Acyl-CoA Elongase 6
AMPK	5′-Adenosine monophosphate-activated protein kinase
GLUT1	Glucose Transporter 1
PFK2	Phosphofructokinase 2
PDH	Pyruvate Dehydrogenase Complex
PDK	Pyruvate Dehydrogenase Kinase
GLS	Glutaminase
ETC	Electron Transport Chain
ROS	Reactive Oxygen Species
