# Supplementary material for: Caffeic Acid Expands Anti-Tumor Effect of Metformin in Human Metastatic Cervical Carcinoma HTB-34 Cells: Implications of AMPK Activation and Impairment of Fatty Acids De Novo Biosynthesis
Source: Int J Mol Sci. 2017 Feb 21;18(2):462. doi: 10.3390/ijms18020462 (PMC5343995; doi:10.3390/ijms18020462)
Supplement: Supplementary file 1 [file ijms-18-00462-s001.pdf]

Supplementary Materials: Caffeic Acid Expands Anti-Tumor Effect of Metformin in Human Metastatic Cervical Carcinoma HTB-34 Cells: Implications of AMPK Activation and Impairment of Fatty Acids De Novo Biosynthesis

Malgorzata Tyszka-Czochara, Pawel Konieczny and Marcin Majka

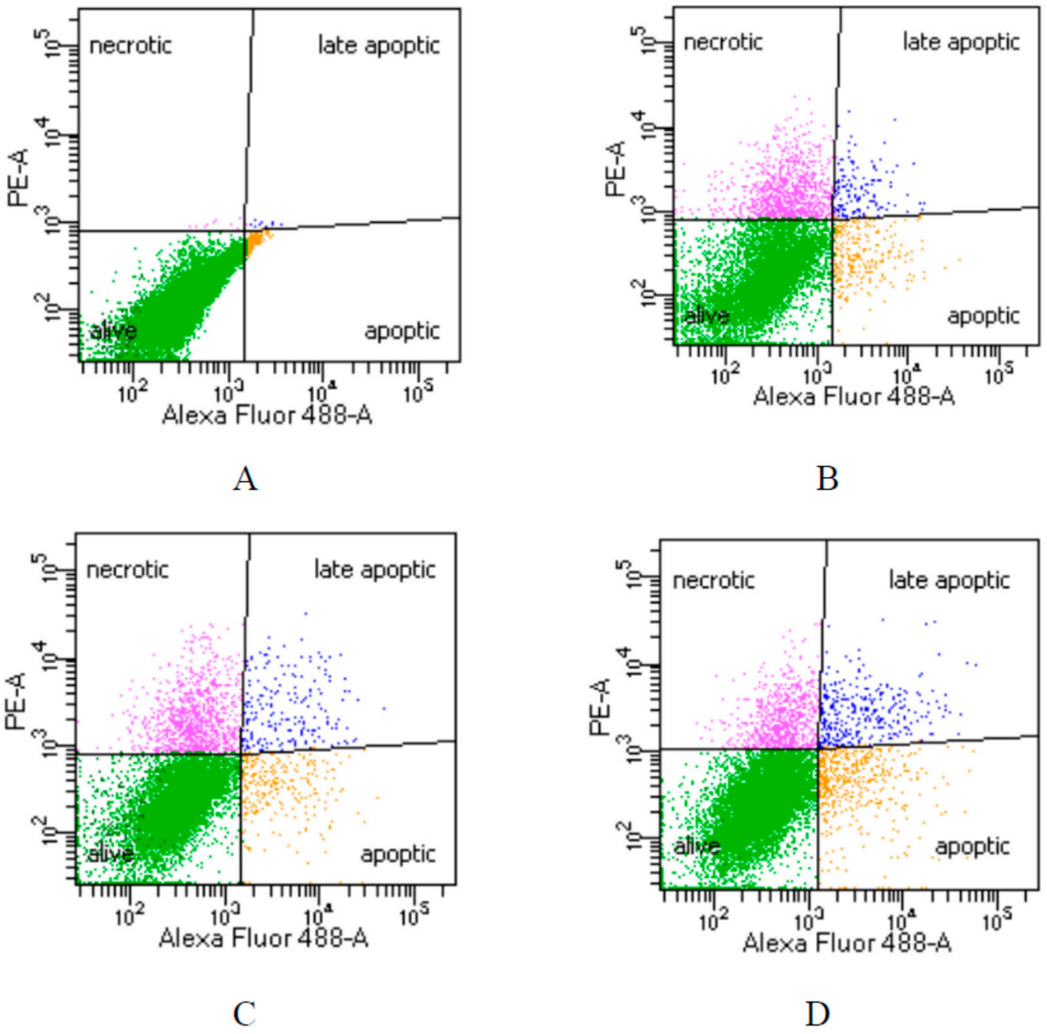

Figure S1. Induction of apoptosis/necrosis in HTB-34 human cervical cancer cells exposed to Met at concentration of 10 mM, CA at 100 µM and Met/CA for 24 hours. The representative dotplots show populations of early/late apoptotic and necrotic cells in untreated HTB-34 cells (**A**), cells exposed to Met (**B**), CA (**C**) and Met/CA (**D**). The cells were stained with Annexin-V (excitation/emission 490/515 nm) and Ethidium homodimer (EthD-III, excitation/emission 528/617 nm) and gated according to forward (FSC), side scatter (SSC) and appropriate fluorescence parameters. According to manufacturer's protocol the alive cells were defined as negative for Anexin-V and EthD-III, the apoptotic cells population consisted of Anexin-V positive/EthD-III negative cells (early apoptosis) and Anexin-V/EthD-III positive cells (late apoptosis); the necrotic cells were Anexin-V negative and EthD-III positive. Additionally, SYTO 41 Blue Fluorescent Nucleic Acid Stain was used to correct discrimination between cells and debris (excitation/emission 483/503 nm). Measurements were performed using FACSCanto10C flow cytometer, with BD FACSCanto System Software (BD Biosciences Immunocytometry Systems, USA).

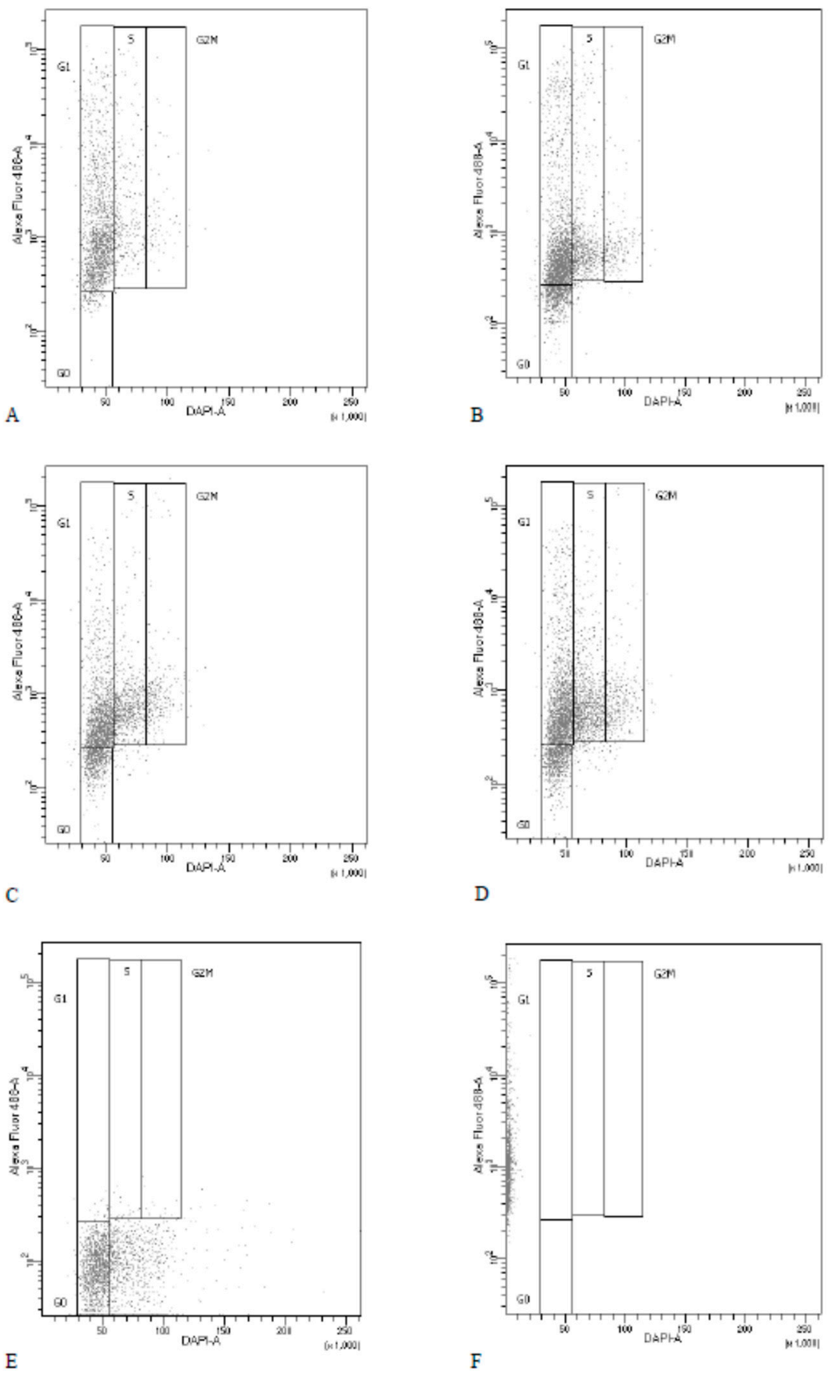

Figure S2. Effects of Met (10 mM), CA (100 μM) and Met/CA on cell cycle distribution in HTB-34 human cervical cancer cells. The cells were exposed to Met and CA for 24 hours, harvested, incubated with KI67 antibody and stained with DAPI (excitation/emission 360/460 nm), The representative dotplots show populations of cells in G0, G1, S and G2/M phase of cell cycle in untreated cells (**A**) and after treatment of Met (**B**), CA (**C**) and Met/CA (**D**). Appropriate control dotplots for DAPI staining without KI67 antibody (**E**) and KI67 without DAPI staining (**F**) were also included. Measurements were performed using FACSCanto10C flow cytometer, with BD FACSCanto System Software (BD Biosciences Immunocytometry Systems, USA).
